# Disease-Modifying Therapy in Multiple Sclerosis: Evaluation of Patients Satisfaction in Iranian Multiple Sclerosis Population

**DOI:** 10.22088/cjim.14.1.89

**Published:** 2023

**Authors:** Negar Molazadeh, Mohammad Ali Sahraian, Mahsa Ghajarzadeh

**Affiliations:** 1 Multiple Sclerosis Research Center, Neuroscience Institute, Tehran University of Medical Sciences, Tehran, Iran; 2 Universal Council of Epidemiology (UCE), Universal Scientific Education and Research Network (USERN), Tehran University of Medical Sciences, Tehran, Iran

**Keywords:** Multiple sclerosis, Satisfaction, Disease-Modifying Therapies

## Abstract

**Background:**

Medication satisfaction is a patient-reported outcome which could show medication adherence. The aim of this study was to determine Iranian MS patients’ satisfaction with Disease Modifying Therapies (DMTs).

**Methods:**

A standardized questionnaire was developed using Treatment Satisfaction Questionnaire for Medication (TSQM). The online link was released on IMSS (Iranian Multiple Sclerosis Society) social media channel, accessible to 4272 MS patients totally.

**Results:**

Three hundred and ninety-four patients participated in our survey with 324 females, 70 males and an F/M ratio of 4.6:1. The most frequent DMTs used were interferon-beta (IFNβ) followed by rituximab. The mean effectiveness and global satisfaction scores were significantly higher for injectable DMTs, while the convenience score was significantly higher for oral DMTs. Mean effectiveness and side-effect scores were significantly higher in the Tysabri group and convenience score was significantly higher in the fingolimod group while global satisfaction was higher in the IFNβ group.

**Conclusion:**

The global satisfaction and effectiveness were significantly higher with injectable DMTs while the convenience score was significantly higher with oral DMTs.

Multiple sclerosis (MS) is a chronic inflammatory disease of the central nervous system with an unpredictable course (1). A wide range of medications known as Disease Modifying Therapies (DMTs) have been approved for the treatment of MS in the recent decade. The balance between efficacy, side effects, and the long-term impact of treatment should be considered through the decision-making process of selection or switching DMTs (2). Higher adherence to prescribed DMTs enhances the level of disease control and may prevent relapse occurrence in MS patients (3). Factors affecting the level of treatment adherence include patient preference and satisfaction, convenience, side effects, and social and economic issues (4). Barbosa et al identified a significant association between the satisfaction of the treatment and compliance and persistence to that treatment (5). Medication satisfaction is a patient-reported outcome (PRO) that reflects the quality of pharmaceutical products and services and could be used as a screening tool for treatment adherence (6). Patient satisfaction with medication which includes items such as effectiveness, convenience, side effects, and global satisfaction provides information regarding the patient’s perception of current treatment. Satisfaction is an important indicator of treatment adherence in patients (5). Iran is a high-risk zone of MS with an increasing incidence of MS in recent years and the number of patients increased dramatically (7). However, no studies have been performed to evaluate drug satisfaction and compare it among various DMTs in Iranian MS patients. So, we conducted this study to investigate MS patients’ satisfaction with DMTs. 

## Methods

This is a cross-sectional study conducted in collaboration with the Iranian Multiple Sclerosis Society (IMSS) in August 2020. The IMSS is a member of the MS International Federation (MSIF) which was established in 1991 in Tehran, Iran. The major goals of IMSS are to develop public awareness about MS and to provide financial and social support, alongside healthcare and rehabilitation services for MS patients in Iran (8). IMSS also conducts a nationwide MS registry system since 1999, which provides a large database of Iranian MS patients (8). All patients with a definite diagnosis of MS based on McDonald's criteria that are diagnosed by neurologists in academic hospitals or private clinics are referred to IMSS where they are registered and get specific member code for receiving support. Registration with IMSS is optional and patients can subscribe the social media channel of this association if they wish to be informed about the latest news and education related to MS.

**Study Objectives**: The primary objective of this study was to evaluate the level of treatment satisfaction in terms of availability, effectiveness, adverse events and convenience in patients with MS taking DMTs. We also aim to find out the association between treatment satisfaction and DMTs adherence status in our patients. Other objectives of our study were to compare the satisfaction extent in patients with different types of DMTs and various routes of administration, including injection, infusion, and oral routes. We also aim to figure out the patients’ perception of treatment benefits and to assess the effect of adverse events on their physical and mental functional status.

**Data Collection:** Data were collected through a standardized questionnaire developed using the valid and reliable Persian version of the Treatment Satisfaction Questionnaire for Medication (TSQM) (9). The online link was released on IMSS social media channel, accessible for 4272 MS patients totally. At the beginning of the questionnaire, patients were asked to answer questions only if they consented to participate in this survey. The answers were blinded and researchers did not have access to patients’ personal information. Patients volunteered to respond to our questionnaire on demographic information including sex, age, marital and occupational status, MS-related data including MS duration and current DMT type, and overall satisfaction with treatments. Satisfaction status in terms of drug availability, costs, and post-marketing support services was assessed using a 3-point Likert Scale where, 1: satisfied, 2: neither satisfied nor dissatisfied, and 3: dissatisfied. Level of satisfaction in terms of effectiveness and convenience was measured using a pivoted 7-point Likert Scale where 1 indicates “completely dissatisfied”, 2 indicates “very dissatisfied”, 3 indicates “dissatisfied”, 4 indicates “almost satisfied”, 5 indicates “satisfied”, 6 indicates “very satisfied” and 7 indicates “completely satisfied”. The effect of adverse events on patient’s satisfaction, functional performance, and mental condition was evaluated using a pivoted 5-point Likert Scale where 1 indicates “very high”, 2 indicates “almost high”, 3 indicates “to some extent”, 4 indicates “very low” and 5 indicates “not at all”. The obtained scores were summed and the percentage of satisfaction score was calculated. 

**Statistical Analysis:** The statistical analysis was performed using SPSS software version 23 (SPSS Inc., Chicago, IL, USA). Data were presented as Mean± SD for continuous or frequencies for categorical variables. A p-value less than 0.05 was considered significant.

## Results

Three hundred and ninety-four patients participated in our survey with 324 females, 70 males and an F/M ratio of 4.6:1. The most frequent DMT used were interferon-beta (IFNβ) followed by rituximab with the level of satisfaction with access to medications and post-marketing support services of 82% and 62% respectively (Table 1). 

**Table 1.** Basic characteristics of the enrolled patients

Variables	N (%)
**Sex** **Female** **Male**	324(82.2%) 70(17.8%)
**Marital status** **Married** **Single**	245(62.2%) 149(37.8%)
**Occupation status** **Employed** **Unemployed**	143(36.3%) 251(63.7%)
**Type of DMT** **Interferon-Beta (IFNβ)Teriflunomide (TFL)** **Dimethyl fumarate (DMF)** **Rituximab (RTX)** **Fingolimod** **Glatiramer acetate (GA)** **Natalizumab (Tysabri)** **Ocrelizumab (OCR)** **Azathioprine (AZA)/ Prednisolone**	125(31.7%) 9(2.3%) 42(10.7%) 113(28.7%) 55(14%) 36(9.1%) 9(2.3%) 2(0.5%) 3(0.8%)

**Figure 1:**
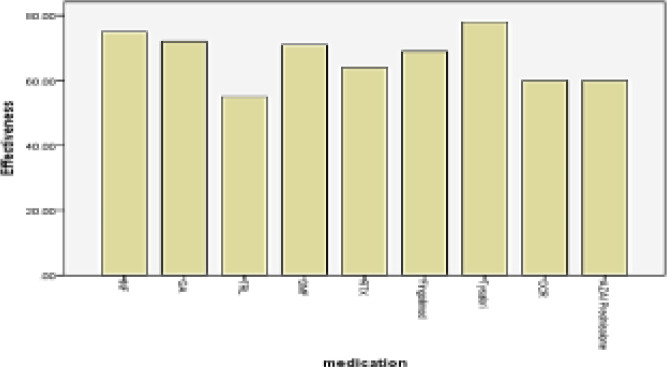
Mean effectiveness score of different DMTs

In total 73.4% of patients were satisfied with the costs of treatments (Table 2). The mean effectiveness and global satisfaction scores were significantly higher for injectable DMTs, while convenience score was significantly higher with oral DMTs (Table 3, figure 1, 4). 

**Figure 2:**
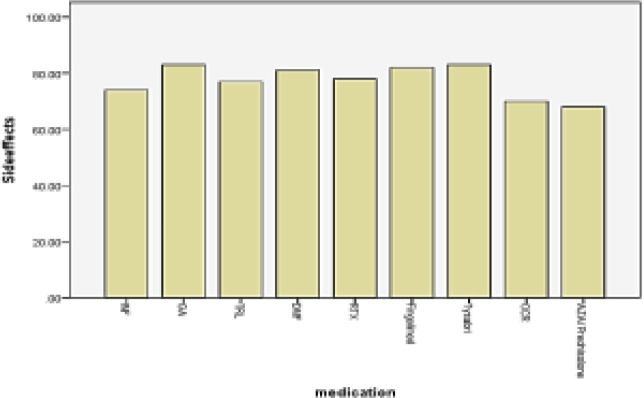
Mean side effects score of different DMTs

**Figure 3:**
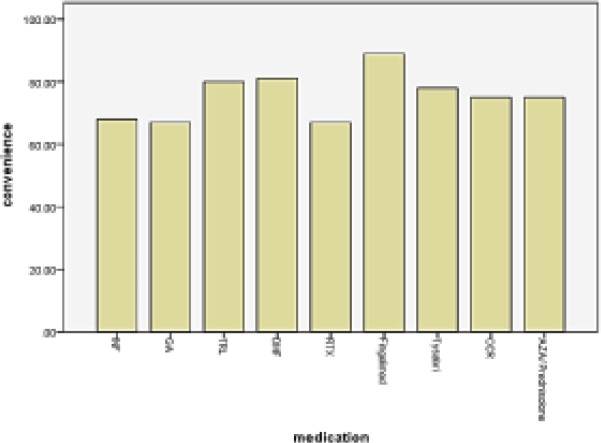
Mean convenience score of different DMTs

Mean effectiveness and side-effects scores were significantly higher in the Tysabri group (figure 1, 2) and convenience score was significantly higher in the fingolimod group, while global satisfaction was higher in the IFNβ group (Table 4, figure 3).

**Table 2:** Patients’ satisfaction with DMTs

Satisfaction Items	N (%)
**Are you satisfied with access to your medication?** **Yes** **No** **No idea **	323 (82%) 33 (8.4%) 38 (9.6%)
**Are you satisfied with the medication cost?** **Yes** **No** **No idea**	289(73.4%) 55(14.2%) 49(12.4%)
**Are you satisfied with the supportive system of your medication company?** **Yes** **No** **No idea**	247(62.7%) 39(9.9%) 108(27.4%)
**TSQM Domains** **Effectiveness** **Side effects** **Convenience** **Global satisfaction**	70.1±20.5 78.4±16.5 73±21.9 57.1±18

**Table 3:** Mean scores of TSQM domains based on administration routes

TSQM Domains	Oral	Injection	Infusion	P value
**Effectiveness**	68.6±18.4	74.6±19.2	65.7±22.7	0.02
**Side effects**	81.2±16.7	76.4±16.2	78.6±16.4	0.07
**Convenience**	85.1±15.9	68.2±21.8	68.9±22.7	<0.001
**Global satisfaction**	65.9±20.2	69.4±19.2	62.5±22.9	0.02

**Figure 4:**
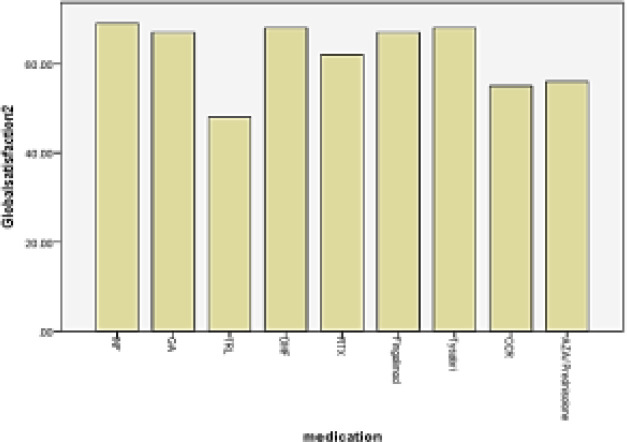
Mean global satisfaction score of different DMTs

**Table 4:** Comparison of TSQM domains in different DMT groups

Type of DMT	Effectiveness	Side effects	Convenience	Global satisfaction
**IFNβ**	75.32±18.90	74.53±16.76	68.52±21.84	69.84±19.20
**GA**	72.36±20.61	83.08±12.49	67.14±20.34	67.87±19.86
**TFL**	55.55±17.75	77.77±20.01	80.83±18.81	48.36±11.28
**DMF**	71.42±17.54	81.70±16.68	81.73±16.18	68.78±20.86
**RTX**	64.77±22.92	78.42±16.54	67.79±23.43	62.18±23.51
**Fingolimod**	69.18±19.02	82.34±15.88	89.05±15.22	67.10±20.16
**Tysabri**	78.88±17.09	83.33±16.77	78.12±17.10	68.94±15.76
**OCR**	60.00±21.21	70.00±7.07	73±21.8	55.10±12.30
**AZA/Prednisolone**	60.00±.00	68.33±25.16	77.50±10.60	56±8.83
**P value**	0.002	0.04	<0.001	0.02

## Discussion

TSQM is designed for assessing treatment satisfaction in chronic diseases such as arthritis, asthma, major depression, type I diabetes, hyperlipidemia, hypertension, migraine, psoriasis as well as MS ()10. Recent studies show that TSQM is useful to study treatment satisfaction with DMTs in patients with relapsing-remitting MS (RRMS) , and the results of the current study demonstrated that global satisfaction and effectiveness were significantly higher in the injectable DMTs group while convenience score was significantly higher in the oral DMTs group. In a previous study, Turčáni et al evaluated 417 MS patients and reported higher significant scores of global satisfaction and effectiveness in the infusion group while convenience was non-significantly higher in the oral group (4). They found significant higher global satisfaction and effectiveness scores in the Tysabri group, followed by fingolimod for effectiveness, and teriflunomide for global satisfaction. They reported that their patients were more convenient with teriflunomide followed by interferon beta-1b. Our results also show that global satisfaction was significantly higher in the IFNβ group followed by Dimethyl fumarate and Tysabri. We also found that effectiveness and side-effects scores were significantly higher in the Tysabri group, and convenience score was significantly higher in the fingolimod group.

In Glanz et al study, patients who were under treatment with Tysabri were more satisfied with the ability to treat or prevent the condition by their medication as well as being more convenient in comparison with IM IFNβ-1a (11). Oral medications have a higher convenience score which is expectable as they could be taken at home without hospitalization and consuming the time for infusion. In the TENERE study which was conducted by Vermersch et al (12), using TSQM; patient-reported level of satisfaction was higher with teriflunomide compared with injectable DMTs (IFNβ and GA) which is in agreement with Turčáni et al study (4). Hanson et al assessed satisfaction in MS patients receiving fingolimod (13) and reported the mean scores of convenience, effectiveness, and global satisfaction of 71.7, 70.1, and 68.9, respectively while mentioned scores were 89, 69, and 67 in our study. Fernández O conducted a study in Spain and evaluated medication satisfaction in MS cases who were under treatment with injectable DMTs and reported the mean scores of convenience, effectiveness and global satisfaction as 72.5, 66.8 and 68.8, respectively (14).

Since availability and costs of treatments, and physician preferences differ among nations, it is crucial to conduct national studies to evaluate medication satisfaction among patients of different countries. Tysabri is administered monthly and our results show that patient satisfaction regarding side-effects is better in Tysabri group when compared to other DMTs. The frequency of side effects plays an important role in determining the degree of medication satisfaction score. For instance, injection site reaction is common in subcutaneous IFNβ and is one of the main causes of switching DMTs in MS patients (15). This study had some limitations. First, it was a single center study. Second, we did not evaluate adherence simultaneously. Larger multi centric studies evaluating adherence and its association with satisfaction is 

In conclusion, the global satisfaction and effectiveness were significantly higher with injectable DMTs, while convenience score was significantly higher with oral DMTs. 

## References

[1] Ghajarzadeh M, Sahraian MA, Fateh R, Daneshmand A (2012). Fatigue, depression and sleep disturbances in Iranian patients with multiple sclerosis. Acta Med Iran.

[2] Montalban X, Gold R, Thompson AJ (2018). ECTRIMS/EAN guideline on the pharmacological treatment of people with multiple sclerosis. Mult Scler.

[3] Thach AV, Brown CM, Herrera V (2018). Associations between treatment satisfaction, medication beliefs, and adherence to disease-modifying therapies in patients with multiple sclerosis. Int J MS Care.

[4] Turčáni P, Mašková J, Húska J (2020). Real-world treatment patterns of disease modifying therapy (DMT) for patients with relapse-remitting multiple sclerosis and patient satisfaction with therapy: results of the non-interventional SKARLET study in Slovakia. Patient Prefer Adherence.

[5] Barbosa CD, Balp MM, Kulich K, Germain N, Rofail D (2012). A literature review to explore the link between treatment satisfaction and adherence, compliance, and persistence. Patient Prefer Adherence.

[6] Atkinson MJ, Kumar R, Cappelleri JC, Hass SL (2005). Hierarchical construct validity of the treatment satisfaction questionnaire for medication (TSQM version II) among outpatient pharmacy consumers. Value Health.

[7] Eskandarieh S, Molazadeh N, Moghadasi AN, Azimi AR, Sahraian MA (2018). The prevalence, incidence and familial recurrence of multiple sclerosis in Tehran, Iran. Mult Scler Relat Disord..

[8] Eskandarieh S, Sahraiain MA, Molazadeh N, Moghadasi AN (2019). Pediatric multiple sclerosis and its familial recurrence: A population based study (1999-2017). Mult Scler Relat Disord..

[9] Shahrbabaki ME, Ahmadipour H, Yousefi N, Divsalar P Treatment Satisfaction Questionnaire for Medication (TSQM Version II): a psychometric properties analysis. ResearchSquare 2021.

[10] Atkinson MJ, Sinha A, Hass SL (2004). Validation of a general measure of treatment satisfaction, the Treatment Satisfaction Questionnaire for Medication (TSQM), using a national panel study of chronic disease. Health Qual Life Outcomes.

[11] Glanz BI, Musallam A, Rintell DJ (2014). Treatment satisfaction in multiple sclerosis. Int J MS Care.

[12] Vermersch P, Hobart J, Dive-Pouletty C (2017). Measuring treatment satisfaction in MS: Is the Treatment Satisfaction Questionnaire for Medication fit for purpose?. Mult Scler.

[13] Hanson KA, Agashivala N, Stringer SM, Balantac Z, Brandes DW (2013). A cross-sectional survey of patient satisfaction and subjective experiences of treatment with fingolimod. Patient Prefer Adherence.

[14] Fernández O, Duran E, Ayuso T (2017). Treatment satisfaction with injectable disease-modifying therapies in patients with relapsing-remitting multiple sclerosis (the STICK study). Plos One.

[15] Beer K, Müller M, Hew-Winzeler AM (2011). The prevalence of injection-site reactions with disease-modifying therapies and their effect on adherence in patients with multiple sclerosis: an observational study. BMC Neurol.

